# Bilateral cellular flows display asymmetry prior to left–right organizer formation in amniote gastrulation

**DOI:** 10.1073/pnas.2414860122

**Published:** 2025-02-03

**Authors:** Rieko Asai, Shubham Sinha, Vivek N. Prakash, Takashi Mikawa

**Affiliations:** ^a^Cardiovascular Research Institute, University of California, San Francisco, CA 94158; ^b^Kumamoto University, International Research Center for Medical Sciences, Kumamoto 860-0811, Japan; ^c^Department of Physics, University of Miami, Coral Gables, FL 33146; ^d^Department of Biology, University of Miami, Coral Gables, FL 33146; ^e^Department of Marine Biology and Ecology, University of Miami, Miami, FL 33149

**Keywords:** L-R asymmetry, gastrulation, bilateral cellular flows, biophysics, particle image velocimetry

## Abstract

A bilateral body plan is predominant throughout the animal kingdom. Bilaterality of amniote embryos becomes recognizable as midline morphogenesis begins at gastrulation, bisecting an embryonic field into the left and right sides, and left–right (LR) asymmetry patterning follows. While a series of laterality genes expressed after the LR compartmentalization has been extensively studied, the laterality patterning prior to and at the initiation of midline morphogenesis has remained unclear. Here, through a biophysical quantification in a high spatial and temporal resolution, applied to a chick model system, we show that a large-scale bilateral counterrotating cellular flow, termed “polonaise movements”, display LR asymmetries in early gastrulation. This cell movement starts prior to the formation of the primitive streak (PS) (the earliest midline structure) and the subsequent appearance of Hensen’s node (the LR organizer). The cellular flow speed and vorticity unravel the location and timing of the LR asymmetries. The bilateral flows displayed a Right dominance after 6 h since the start of cell movements. Mitotic arrest that diminishes PS formation resulted in changes in the bilateral flow pattern, but the Right dominance persisted. Our data indicate that the LR asymmetry in amniote gastrula becomes detectable earlier than suggested by current models, which assume that the asymmetric regulation of the laterality signals at the node leads to the LR patterning. More broadly, our results suggest that physical processes can play an unexpected but significant role in influencing LR laterality during embryonic development.

## Significance

Bilaterians are defined by a bilaterally symmetrical body plan. Vertebrates exhibit external bilateral symmetry but display left–right (LR) asymmetry in their internal organs. Amniote embryos switch the patterning of internal organs from bilateral symmetry to LR-asymmetry. Using chick embryos as a model system, here we examined the initiation of LR symmetry breaking. Our biophysical approaches to quantify cellular flows inferred that LR symmetry breaking occurs before the formation of Hensen's node, a LR organizer, which serves as a signaling center for LR patterning-gene programs. Our work demonstrates that quantitative biophysical parameters can help unravel the initiation of LR symmetry breaking, suggesting an involvement of physical mechanisms in this critical biological patterning process.

Most animals are classified in the *Bilateria*, possessing a morphological symmetry between the left–right (LR) sides conferred by the midline. Vertebrates present a complex bilateral body plan, displaying bilateral symmetry externally but with asymmetry in the internal organs (1–3). LR patterning and morphogenesis have been extensively studied for events postformation of Hensen's node in amniotes (Kupffer's vesicle in fish)(4, 5), which induces the asymmetric expression and regulation of the LR regulatory genes (6, 7).

In amniote embryos, the bilateral body plan becomes recognizable at gastrulation during midline morphogenesis, compartmentalizing the embryonic field into the left and right sides (7, 8). The primitive streak (PS) is the earliest midline recognizable landmark and serves as a signaling center of gastrulation (9, 10). Prior to and during PS extension, a counterrotating cellular flow, termed “polonaise movements”, appears at both LR sides along the midline axis (11–17). This bilateral rotating cellular flow continues until ingression to generate germ layers starts, at the midline through the PS. At this point, the cellular flow shifts from a rotation pattern to a lateral-to-medial pattern toward the midline or PS (18). Previously, we have shown that the lateral-to-medial movements of the epiblast demonstrate ipsilaterality, or side-identity, along the PS (19). Furthermore, a LR-asymmetric membrane potential within the extending PS has been reported (20). These studies hint at a possibility that LR laterality might arise even before the genetically programmed LR patterning is established.

Here, we examine the earliest timing of LR asymmetry patterning in amniote gastrulation. Utilizing computational analyses based on physics of fluids (21), we examined the cellular flows associated with the polonaise movements during early gastrulation in the chick embryo. Visual mapping of biophysical parameters of cellular flows over time revealed that the cellular flows on the right side were dominant than in the left during PS formation. To test the role of midline structure, PS formation was diminished through mitotic arrest. We show that the resulting embryos displayed a shortened duration of bilateral cellular flows and ended with total right-side dominance of the cellular flows. These data suggest that the laterality program regulating the right-side dominance of the cellular flows is already set up prior to both maturation of the midline and LR asymmetric expression of the well-known laterality genes at Hensen’s node, and it does not depend on cell division.

## Results

### Bilateral Flows During Early Midline Morphogenesis in Chick Gastrulation.

**Fig. 1.**
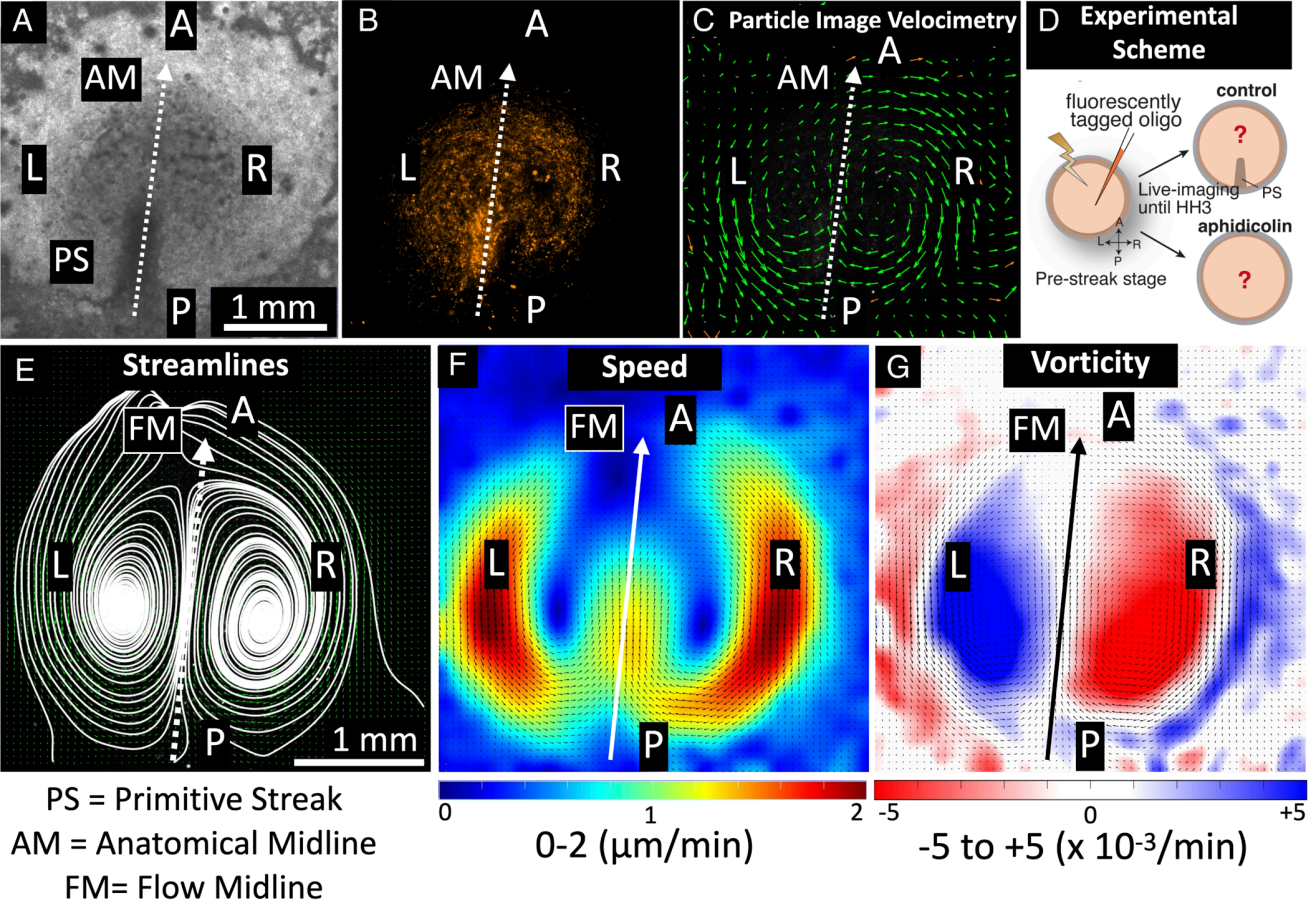
Cellular flows during early chick embryo development. *Upper* panel: (*A*) Brightfield image of chicken embryo gastrulation showing development of the primitive Streak (PS) at the midline (at stage HH3). The anatomical midline (AM) defined by the PS is shown in dotted lines, and the anterior (A), posterior (P), *Left* (L), and *Right* (R) regions are also labeled. The images shown in (*A*–*C*) correspond to the time point of 10 h post initiation of cellular motion in control sample 1 (Table 1). (*B*) Fluorescence image of the embryo revealing cells tagged with fluorescent markers. (*C*) Results from Particle Image Velocimetry (PIV) analysis (*Materials and Methods*), the green arrows represent instantaneous velocity vector fields. (*D*) A schematic showing the experimental scheme (*Materials and Methods*). *Lower* panel: (*E*–*G*) Time-averaged (over 10 h) results from PIV analysis. FM is the flow midline (Fig. 4). (*E*) Streamlines reveal bilateral cellular flow patterns with counterrotating vortices in both L and R regions. (*F*) Cellular speeds: The heat map indicates lowest speeds in blue color and highest speeds in red color. (*G*) Vorticity (measurement of rotation): Red colors indicate clockwise rotation, and blue colors indicate counterclockwise rotation directions. Both speed and vorticity plots show slightly higher magnitudes on the *Right* side, indicating asymmetry in the bilateral flows (*F* and *G*). The length scale bar shown in (*A*) is the same for panels (*A*–*C*), and length scale bar in (*E*) is the same for panels (*E*–*G*).

To survey the timing of initiation and patterning of the bilateral cellular flow, we performed live-imaging experiments during the early chick gastrulation process, from prestreak stage until HH3 [Hamburger and Hamilton staging (22)] (Fig. 1 *A*, *B*, and *D*) (*Materials and Methods*). To quantitatively analyze the cellular flow during early midline morphogenesis or PS formation, the image sequences of fluorescently tagged cells (*Materials and Methods*) were postprocessed using the particle image velocimetry (PIV) method (21, 23). PIV is a combined experimental and computational technique from fluid mechanics that is used to quantify time-varying fluid velocity fields by analyzing time-lapse datasets with moving tracer particles (*Materials and Methods*). A snapshot of the flow velocity vectors is shown in Fig. 1*C*, indicating the presence of the well-known polonaise movements in our experiments (Movie S1).

To obtain a comprehensive pattern of the bilateral cellular flows, time-averaged data of the flow fields were generated for the entire imaging duration (10 h) (Fig. 1 *E*–*G*) (*Materials and Methods*). The time-averaged flow streamlines revealed the intricate structure of two counterrotating vortices that characterize the bilateral cellular flows (Fig. 1*E*). The time-averaged speed calculations showed that the fastest cellular flows were in the peripheral regions of the vortices, on both L and R sides (Fig. 1*F*). The bilateral flows also had a local rotational component that was quantified using a metric known as vorticity. We extracted the time-averaged vorticity information, and the two counterrotating vortices were visible in L and R sides (blue- and red-colored regions, respectively) (Fig. 1*G*). The darker colored regions in L and R (blue- and red-colored, respectively) indicate highest vorticity magnitudes, corresponding to the location of the bilateral vortices (Fig. 1*G*). The vorticity values considered for further quantification were only those lying inside the embryonic region (*SI Appendix*, Fig. S1).

Strikingly, the full time-averaged analysis provided higher-resolution data for spatiotemporal patterning of the bilateral cellular flows. Looking closely at the speed plot (Fig. 1*F*), the regions with higher speed on the R side (yellow and red regions) qualitatively occupy a slightly larger area than on the L side and quantification of this area is carried out in sections that follow. Similarly, the vorticity regions in R (red) qualitatively occupied a larger area than the L (blue) (Fig. 1*G*). These time-averaged results suggest that the bilateral flows may not be fully symmetric. A LR asymmetry, more specifically a R dominance, became evident (Fig. 1 *F* and *G*).

### Temporal Changes in Bilateral Flows During Early Midline Morphogenesis in Chick Gastrulation.

**Fig. 2.**
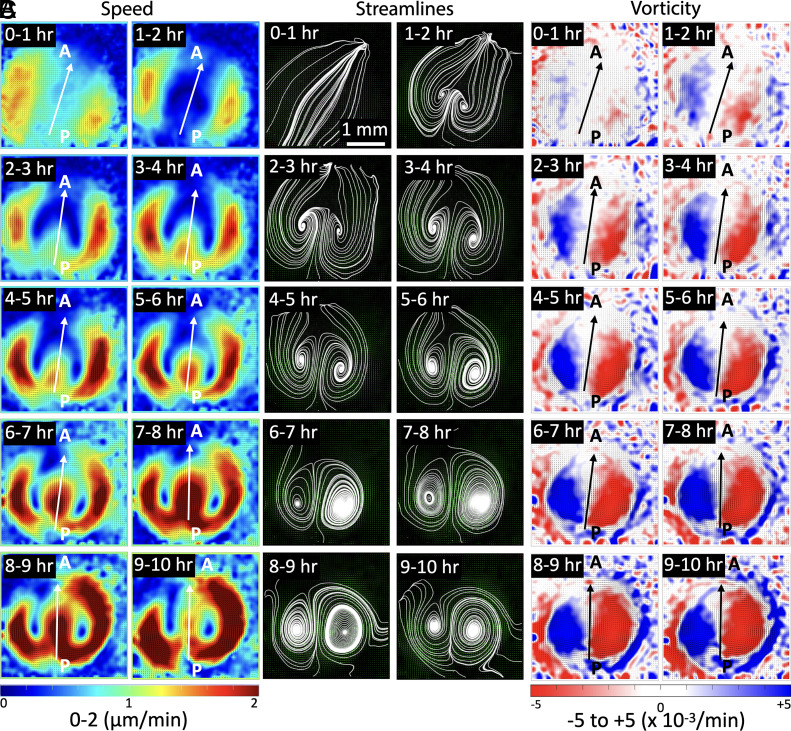
Temporal progression of bilateral flows during early chick embryo development. Short time-averaged (1 h) sequences of (*A*) speed, (*B*) streamlines, and (*C*) vorticity from PIV analysis of control sample 1 (Table 1). (*A*) Speed: cellular flows show initial speed transients (0 to 2 h), which later stabilize and lead to symmetric L-R speed patterns (2 to 7 h). This symmetry in flow speed is lost at the later stages (7 h onward) and cellular speeds become R dominant. (*B*) Streamlines: Cellular flows initiate only at the sides (0 to 1 h), and then progress in the midline region displayed by the bilateral transients (1 to 3 h). Later these bilateral flows form two stable counterrotating vortices (3 h onward). (*C*) Vorticity: Initial flow transients (0 to 2 h) stabilize to form symmetric bilateral flows (2 to 7 h) with well-defined counterrotating vortices (blue-red colors). Following this (7 h onward), the L-R symmetry is lost, with the R vortex being dominant. The arrows represent the flow midlines (FM) and indicate the direction of flows at that location (*Materials and Methods*). The length scale bar shown in the first streamline panel (0 to 1 h) is the same for all panels.

To study the time-varying bilateral cellular flow patterns in the L and R, we performed the PIV averaging by generating hourly time-averaged plots for the entire 10 h of imaging (Fig. 2). The cellular flows were accompanied by formation of the two counterrotating vortices (see streamlines, Fig. 2). In the initial hours (0 to 2 h), these vortices were not fully developed. Whereas, at later time points, they formed two fully developed counterrotating vortices (4 h onward) (Fig. 2). The speed plots showed that the cellular flows initiated along the peripheral regions (0 to 2 h) and merged at the posterior end. The flows then proceeded from posterior (P) to anterior (A) along the flow midline (FM) axis (3 h onward) (Fig. 2). In the next 4 h (3 to 7 h), the L and R sides in the speed plots presented high symmetry. At later times (7 to 8 h), the flow on the R side dominates with a larger area containing higher speeds, and this R domination continues till the end. The streamlines also showed that at later times the R vortex becomes larger in size than the L vortex. The vorticity calculations also showed initial unstable transients (0 to 2 h) but stabilized after a few hours (Fig. 2). As the bilateral flows proceeded, the L (blue) and R (red) side vortices grew in size to form stable counterrotating vortices. At a later timepoint (6 h onward), the R (red) vortex was getting slightly larger in area compared to the L (blue) vortex. This R side vorticity dominance further increased and persisted throughout this window (9 to 10 h).

In another dataset (control sample 2), the bilateral flows initially began with a L dominance and at the end transition to R dominant flows (*SI Appendix*, Fig. S2) as seen with other samples. In this study, we define the “dominance” of the cellular flow to explicitly describe that flow strength is larger on one side compared to the other. Table 1 lists the origin and the end dominance of flow in all the four control samples in this study. All the cases exhibited R dominant flow at later times. In summary, during early midline morphogenesis, i.e. PS formation during gastrulation in chick embryos, there was a temporal flow transition from L to R dominance pointing that cellular flows exhibit LR asymmetry.

**Table 1.** Features of bilateral flows in control versus aphidicolin-treated embryos

Sample Number	Origin of flows in L-R, initial dominance	Duration of Bilateral flows	Flows toward end stages in L-R, end dominance	End dominance starting time
Control Sample 1	L	0 to 10 h	R	7 to 8 h
Control Sample 2	L	0 to 10 h	R	7 to 8 h
Control Sample 3	L	0 to 10 h	R	Not clear
Control Sample 4	R	0 to 10 h	R	Always
Aphidicolin Sample 1	R (left vortex forms)	No bilateral flow, vortex direction switches	R	6 to 7 h
Aphidicolin Sample 2	R	No bilateral flow	R	Always
Aphidicolin Sample 3	R	0 to 7 h	R	Always
Aphidicolin Sample 4	R	No bilateral flow	R	Always

**Fig. 3.**
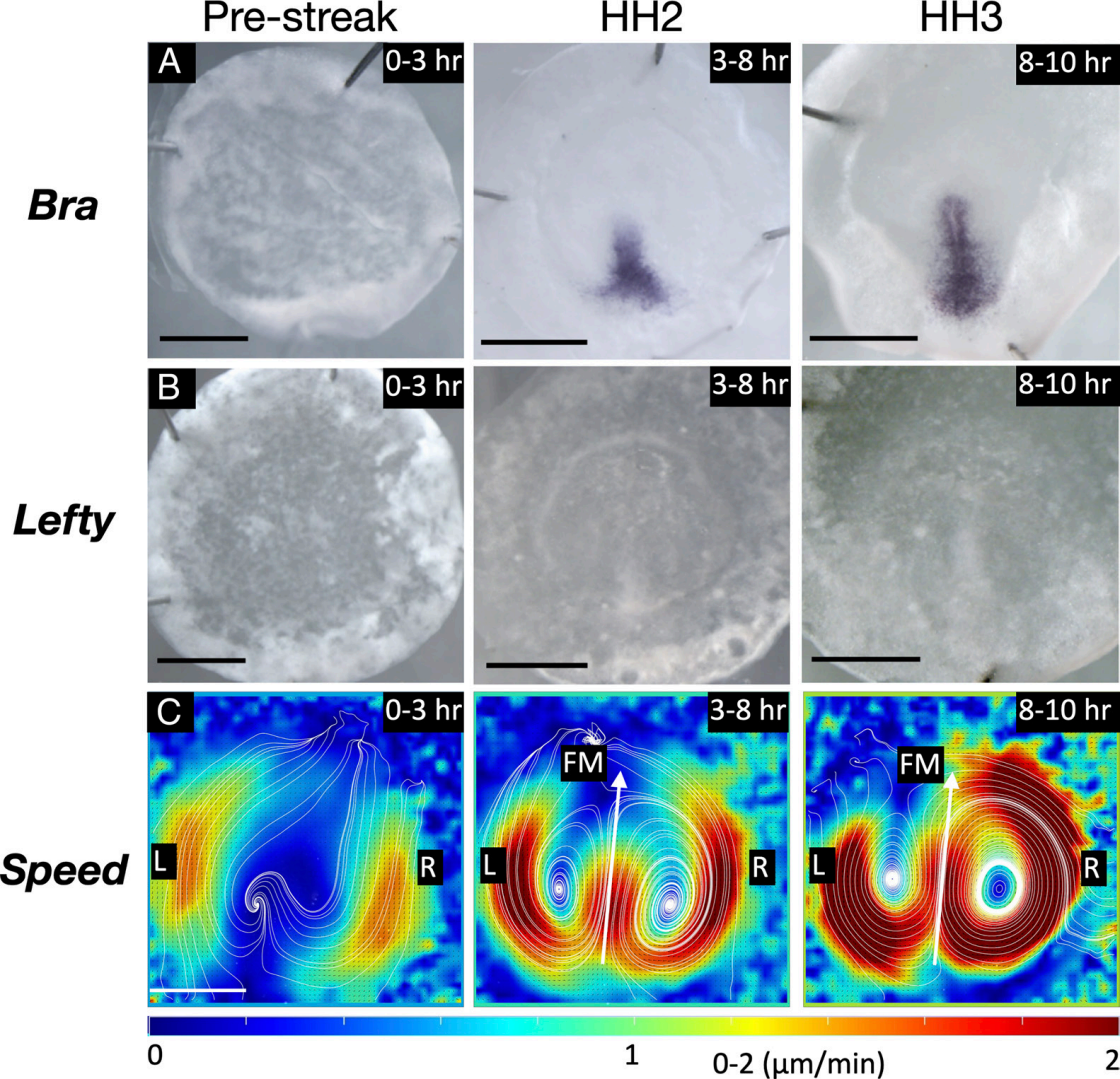
Expression analysis of Lefty (a laterality gene) and the cellular flow. (*A* and *B*) Prestreak stage to HH3 whole-mount in situ hybridization of *Brachyury* (*A*), Lefty (*B*). (*C*) Heat map of time-averaged speed. (Scale bars, 1 mm.)

To test whether known laterality genes are detectable at and during LR asymmetry of cellular flow as early midline morphogenesis proceeds, the expression patterns of well-established LR regulatory genes (e.g., *Shh*, *Lefty*, and *Nodal*) were examined during PS extension by whole-mount in situ hybridization (WISH) (Fig. 3 and *SI Appendix*, Fig. S3, *Materials and Methods*). At prestreak stage X [Eyal-Giladi and Kovchav staging (24)], the expression of *Brachyury* (a PS and early mesodermal cell marker) was not detectable by NBT/BCIP staining (Fig. 3 and *SI Appendix*, Fig. S3). Upon elongation along the midline, PS cells became distinguishable from other embryonic cells as the area of *Brachyury-positive* (a PS and early mesodermal cell marker) and *Sox3-negative* (an epiblast marker) expression (*SI Appendix*, Fig. S3). The PS appears from the posterior marginal zone at HH2 (*SI Appendix*, Fig. S3) and anteriorly extends along the midline (*SI Appendix*, Fig. S3), until the Hensen’s node, which is called as “the LR-organizer” in bird embryos, formed at the anterior tip of the PS at HH4 (22). The NBT/BCIP staining showed that the *Brachyury*-expression within the PS was detectable within the extending PS, while the LR asymmetric expression pattern of the LR regulatory genes became remarkable at Hensen’s node at or after HH4 (*SI Appendix*, Fig. S3), consistent with previous reports (25–27). It must be noted that the classical WISH methods, which have been commonly used in previous (25–27) and the present studies (*Materials and Methods*), have a technical limitation of lower sensitivity than the latest RNA detection techniques, such as hybridization chain reaction (HCR) (28). The bilateral cellular flows display qualitatively symmetric flow at HH2 but exhibit asymmetry with a R dominance at HH3 (Fig. 3*C*). Thus, within the technical limitations of the WISH method, LR asymmetries in the cellular flows were detected prior to the expression of well-established LR regulatory genes (e.g., *Shh*, *Lefty*, and *Nodal*) in distinct LR asymmetric patterns at Hensen's node.

### Biophysical Quantification of Bilateral Flows During Early Midline Morphogenesis.

**Fig. 4.**
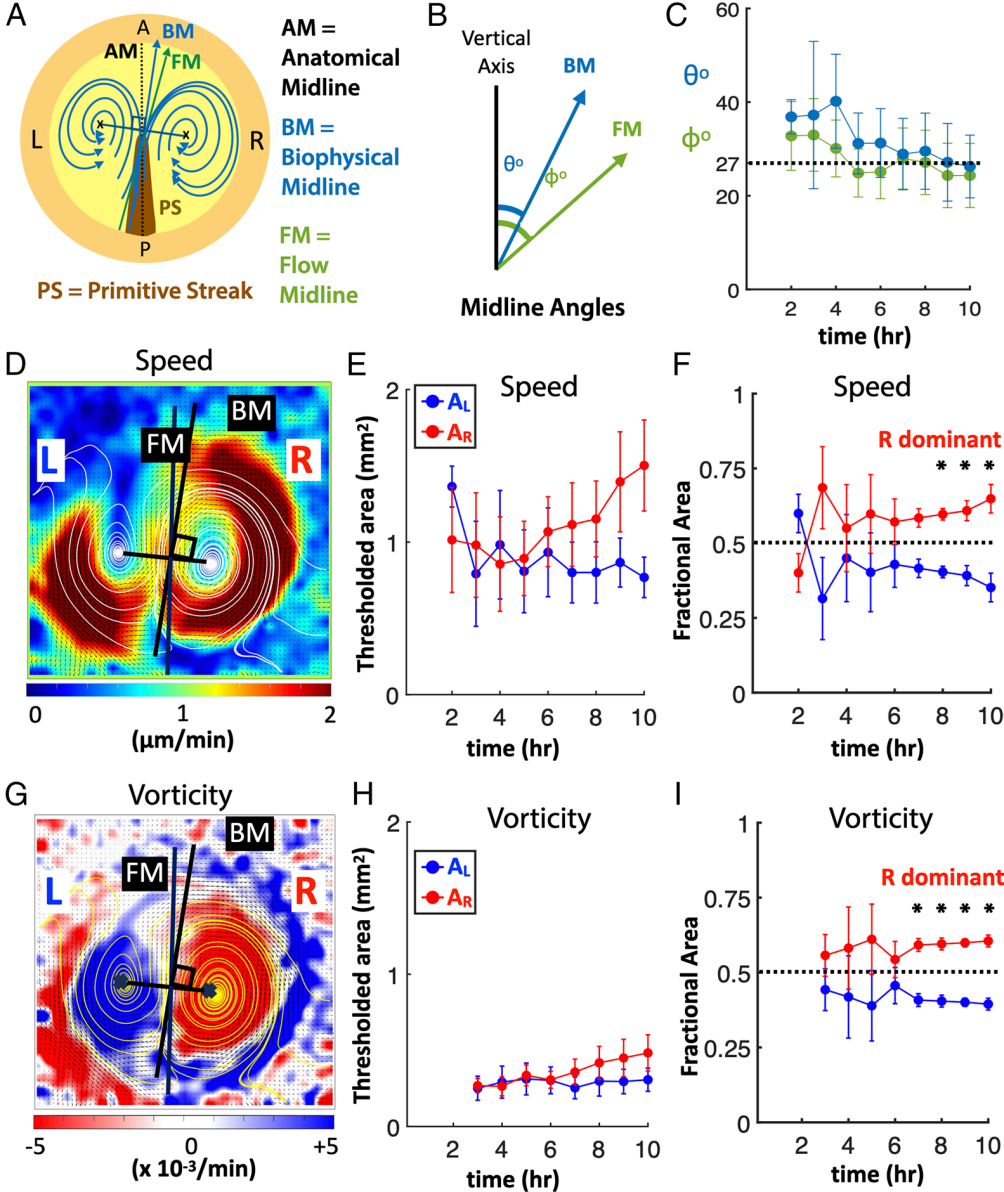
Biophysical quantification of bilateral flows over time. (*A*) Scheme representation of bilateral flows accompanied by primitive streak (PS) formation. The three distinct midline axes are shown: AM, BM, and FM (*SI Appendix*). (*B*) The angle subtended by FM and BM to the vertical axis. (*C*) The averaged (N = 4) mean angles of BM and FM for the control samples. The dotted black line shows the constant angle maintained by AM with the vertical axis. (*D*) Heat map showing cell flow speeds with the L and R regions of the embryo separated by the FM. (*E*) The averaged mean (N = 4) of thresholded area of speeds in L (A_L_) and R (A_R_) regions of control samples. (*F*) The averaged mean (N = 4) of fractional area in L and R regions, corresponding to A_L_/(A_L_+A_R_) and A_R_/(A_L_+A_R_) respectively. (*G*) Heat map showing the vorticity in L and R regions (blue and red respectively). (*H*) The averaged mean (N = 4) of thresholded area of vorticity in L (A_L_) and R (A_R_) regions. (*I*) The averaged mean (N = 4) vorticity fractional area in L and R regions. In (*F*) and (*I*), *represents the *P* value (0.03) obtained from a nonparametric Wilcoxon test (*SI Appendix*). In (*E*), (*F*), (*H*), and (*I*), the results correspond to a 50% threshold (*SI Appendix*). The error bars in all plots represents the SE.

To quantify the degree of LR asymmetry, the data were analyzed on the L and R sides separately along a midline axis. Three possible midline axes were defined: anatomical midline (AM), biophysical midline (BM), and FM. The AM is the line passing through the middle of the PS, and BM is the perpendicular bisector to the line connecting the bilateral vortex centers. The FM is the line parallel to the cellular flow direction in between the bilateral vortices (*Materials and Methods*) (Fig. 4*A* and *SI Appendix*, Fig. S4). If the bilateral vortices were symmetric, these three midlines would coincide with each other. To test this hypothesis, we quantified them over time and measured their angular variation with respect to the vertical (Fig. 4*B* and *SI Appendix*, Fig. S4). The angular separation between BM and FM was larger at the initiation of the bilateral flow but reduced when the vortices were fully developed (Fig. 4*C*). Further, the three midline axes were not coincident with each other throughout the time. These results support the model that the bilateral cellular flows are asymmetric.

In addition to the midline axes, the positional variations in the L and R vortices were detected over time. This positional variation was quantified by measuring two parameters: positions of the vortex centers (*SI Appendix*, Fig. S5) and the distance between the two centers over time (*SI Appendix*, Fig. S6). The X and Y locations of the two vortex centers were tracked over the 10-h duration (*SI Appendix*, Fig. S5). The origin (reference point) was chosen to be outside the embryonic disc. The results did not show any significant shift in the X or Y displacements of the vortex centers (N = 4 control samples). Hence, this analysis demonstrated that there was no systematic drift in any direction due to the embryonic disc or the culture system. The average distance between the vortex centers (N = 4) showed larger fluctuations only during the initial hours when the flow was unstable, and later became smaller showing steady fluctuations when the flow stabilized (*SI Appendix*, Fig. S6 *B* and *C*).

In the analyses that follow, we only retained the cellular bilateral flows inside the circular embryonic region. The extraembryonic material surrounding the embryonic disc was stationary, so our quantification methods gave rise to results that were noise or artifacts, which were discarded (*SI Appendix*, Fig. S1). The cellular flow speed and vorticity in the L and R regions showed R dominance (Figs. 1 and 2). In addition, the thresholded area of cellular flows for both speed and vorticity increased as the embryos developed. These areas on the L and R regions are denoted as A_L_ and A_R_ respectively. The time evolution of A_L_ and A_R_ for speed and vorticity for all samples with three threshold values (30%, 50%, 70%, *SI Appendix*, Fig. S7) are shown in *SI Appendix*, Figs. S8 and S9, and the averaged A_L_ and A_R_ for speed and vorticity (N = 4 samples) are shown in *SI Appendix*, Fig. S10. Fig. 4 *E* and *H* show the hourly averaged mean (N = 4 samples) A_L_ and A_R_ of speed and vorticity in L (A_L_) and R (A_R_) regions and their time evolution (50% threshold value is chosen to be the most representative). For both speed and vorticity, we observed that initially (up to 6 h) A_L_ and A_R_ have similar values. Whereas, at later time points (after 6 h), A_R_ dominates with larger values (Fig. 4 *E* and *H*). This dominance of A_R_ further increases at the end time points.

The embryonic size variations across different samples were considered by calculating the fractional areas of A_L_ and A_R_ regions, i.e., A_L_/(A_L_+A_R_) and A_R_/(A_L_+A_R_) respectively. Fig. 4 *F* and *I* shows the averaged mean (N = 4 samples) fractional area for speed and vorticity regions respectively and its time evolution. A value of 0.5 fractional area represents the line of symmetry (represented by the dotted line in Fig. 4 *F* and *I*). Any deviation of fractional area value from 0.5 represents asymmetricity. In the beginning stages (up to the initial 6 h), the L and R fractional areas are closer (symmetric). But at later time points (after 6 h), the fractional area values separate out with R being dominant (>0.5). The fractional areas showed significant statistical differences in L and R beyond 6 h, with p-values of 0.03 obtained from a nonparametric Wilcoxon test (see *Materials and Methods* and *SI Appendix*). Hence, the bilateral cellular flows showed a clear R dominance at the end stages.

### Bilateral Flows in Cell-Division-Inhibited Embryos During Early Midline Morphogenesis.

**Fig. 5.**
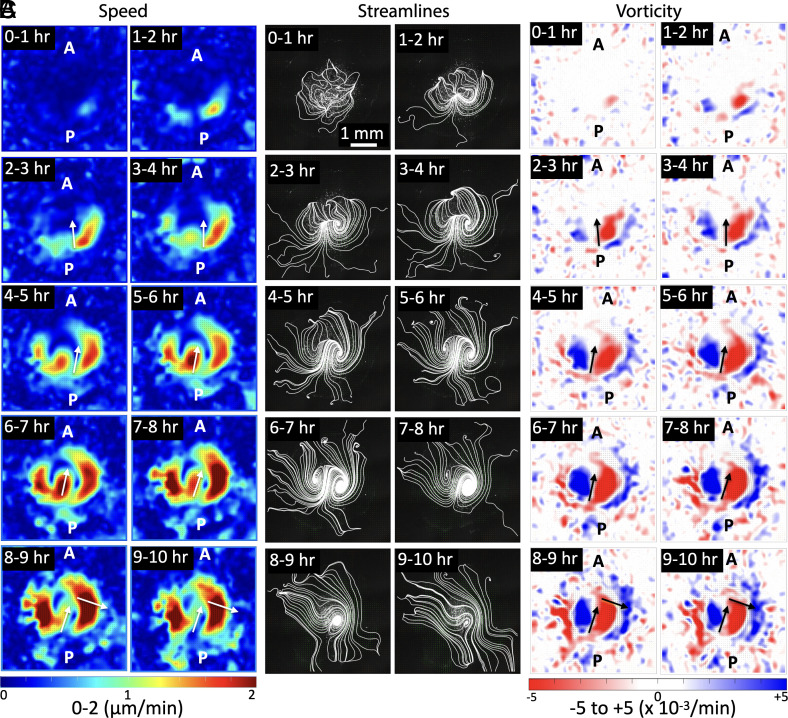
Time evolution of flows in cell-division-inhibited chick embryos. Short time-averaged (1 h) sequences of (*A*) speeds, (*B*) streamlines, and (*C*) vorticity from PIV analysis of aphidicolin sample 3 (Table 1). (*A*) Speed: Cellular flows are initially transient and first start on the R side (0 to 2 h). These transients stabilize without any L-R symmetry with a dominant R side pattern until the end (2 to 10 h). (*B*) Streamlines: The initial cellular flow transients are random (0 to 2 h), but following these bilateral flows develop (2 to 7 h). Subsequently, the flows lose L-R symmetry and turn into a single vortex on the R side (7 to 10 h). (*C*) Vorticity: initial flow transients (0 to 2 h) develop to form bilateral flows (2 to 7 h) with well-defined counterrotating vortices (blue-red colors). After this (7 h onward) even though there is only a R vortex present (red), the blue color indicates the counterclockwise rotational regions that still persist (but do not form a closed vortex) (*Materials and Methods*). The short arrows serve as a guide to indicate direction of flows at that location, not representing the midlines (*Materials and Methods*). The length scale bar shown in the first streamline panel (0 to 1 h) is the same for all panels.

The above results demonstrated R dominance of the bilateral cellular flows during PS extension. Our recent work has shown that the bilateral cellular flow initiates with diminished PS formation under mitotic arrest (i.e. inhibition of cell division) (29). However, it remains unclear whether proper PS formation was required for R dominance of the bilateral cellular flows. To test the question, we performed live-imaging of the early chick gastrulation process under mitotic arrest caused by aphidicolin (a DNA polymerase inhibitor) (Fig. 5, *SI Appendix*, Fig. S11, and Movie S2). When cell division was inhibited, the PS structure was significantly diminished, consistent with our recent study (*SI Appendix*, Fig. S11) (*Materials and Methods*).

Under mitotic arrest, the cellular flow patterns in the PS-diminished embryos displayed different patterns from those seen in the control (Fig. 5 and Movie S2). The flows started with a side dominance (frequently R) (Table 1) forming a bilateral flow briefly (Fig. 5). This bilateral flow later merged into one single R vortex. The initial presence of this bilateral flow had already been lost in some cases (*SI Appendix*, Fig. S12) (Table 1). However, in all cases (N = 4), the flows merged to form a single vortex with a R dominance at later stages. The temporal evolution of the speeds also showed that the cellular flows were always R-dominant (Fig. 5). Similarly, for the vorticity as well, a R dominance was detectable from the initial stages (clockwise rotating vortex region in red color). The blue-colored regions in the vorticity plots correspond to curved flow regions that have counterclockwise rotations, but a closed vortex did not form (confirmed from the corresponding streamlines) (Fig. 5). In these embryos that were under mitotic arrest, PS formation was diminished (*SI Appendix*, Fig. S11), indicating that the AM was not defined in these embryos. Further, consistent BM and FM were not defined, because the bilateral flows did not continue throughout this time window (Fig. 5 and Movie S2). While these technical limitations made it difficult to carry out further biophysical quantification, the above PIV analyses clearly identified that R dominance of the bilateral cellular flows persisted under mitotic arrest.

### Comparison of Bilateral Flows in Control Versus Cell-Division-Inhibited Embryos During Early Midline Morphogenesis.

**Fig. 6.**
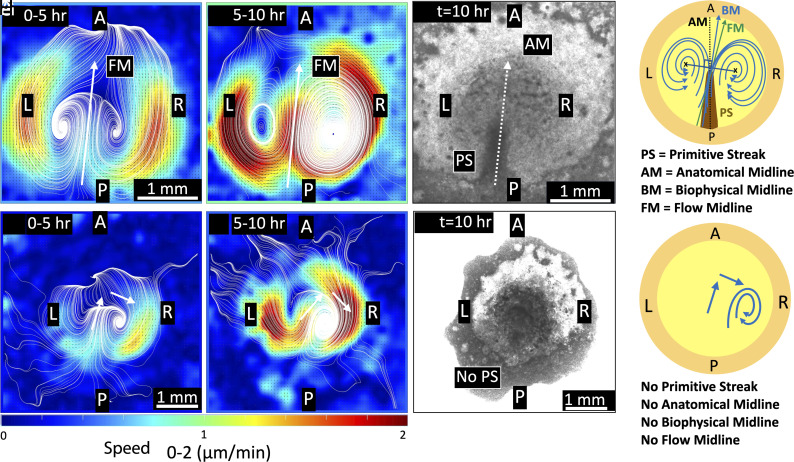
Comparison of cellular flows in early chicken embryo development: control vs cell division inhibited. Time-averaged (5 h) speed results from PIV analysis of the control and aphidicolin-treated embryos (Table 1). *Top* panels – control embryo (control sample 1, Table 1): (*A*) During the initial period (0 to 5 h), the bilateral flows have a high degree of L-R symmetry. (*B*) In the later period (5 to 10 h), the bilateral flows may still be maintained but the L-R symmetry is lost, and the R dominance is visible. (*C*) The brightfield image at the end time point (t = 10 h) showing PS. (*D*) The schematic represents the bilateral cellular flows in the control, with flows dominant in R. The PS forms but the AM and BM axes may have a slight deviation from each other. *Bottom* panels – Cell-division-inhibited embryos (aphidicolin sample 3, Table 1): (*E*) During the initial period (0 to 5 h), there is a single vortex formation on the R side. (*F*) In the later period (5 to 10 h), the single R vortex persists and dominates the flow speeds. (*G*) The brightfield image at the end time point (t = 10 h) with no PS formation. (*H*) The schematic represents the cellular flows in cell-division-inhibited embryos, with single R vortex–dominated flows. The PS did not form, and the midlines AM, BM, and FM were not defined. The short arrows serve as a guide to indicate direction of the flows at that location, not representing the midlines (*Materials and Methods*). The length scale bar in (*A*) is the same for all panels in *Top* row, and length scale bar in (*E*) is same for all panels in *Bottom* row.

The cellular flows in the control and cell-division-inhibited embryos are compared and summarized here. Since biophysical quantification indicated that the degree of LR asymmetry of the cellular flow shifted at approximately 5 h (Fig. 4), we calculated the half-time averages (5 h) of the datasets to account for these temporal flow patterns and variations. These half-time averages capture the flow dynamics during the first half (0 to 5 h) and the second half (5 to 10 h) of the early midline morphogenesis process in both the control and cell-division-inhibited cases, as shown in Fig. 6.

Looking at the half-time averaged cellular flow speeds in the control case, in the first half (0 to 5 h), the flows were symmetric in L and R (Fig. 6*A*). Whereas, in the second half (5 to 10 h), the flows became R dominant (Fig. 6*B*), demonstrating that bilateral flows end with a R dominance during early midline morphogenesis (Fig. 6 *A* and *B*). In these embryos, the bilateral cellular flows occurred during the formation of the PS structure (Fig. 6*C*), and the three distinct midlines (AM, BM, and FM) can be defined, tracked, and quantified.

In the cell division inhibited case, the cellular flows deviated from the classic bilateral mode (Fig. 6 *E* and *F*). R dominance of the cellular flows was recognizable in both the first half (0 to 5 h, Fig. 6*E*) and the second half (5 to 10 h, Fig. 6*F*), with a single R vortex forming toward the end. The three distinct midlines were not defined because of these complex cellular flow patterns and diminished PS formation (Fig. 6*G*) under mitotic arrest. Taken together, R dominance of the bilateral cellular flows persisted even though PS formation was diminished under mitotic arrest.

## Discussion

The present study identified previously undetected LR asymmetries in the bilateral cellular flows (the polonaise movements) during early midline morphogenesis in chick gastrulation. Although the bilateral cellular flows have been studied for approximately a 100 y (10, 11, 30–35), this is the first time LR asymmetries in flow have been identified. Current models assume that the LR laterality in amniotes arises in later stages of development, when the set of the well-known laterality genes triggers asymmetry patterning after the appearance of the node (4, 6, 27, 36–38). An implication, based on the current model, would be that earlier patterning events, including the bilateral cellular flows, are symmetric. However, our results are inconsistent with this assumption. The present study with detailed biophysical quantification techniques and approaches for time-averaging of cell speeds and vorticity reveals that bilateral flows had already exhibited LR asymmetry toward R dominant flow during PS extension and prior to Hensen's node formation. Thus, our unique time-averaging approach to quantify bilateral cellular flows enabled the visualization and quantification of these LR flow asymmetries.

Our present study showed that the LR asymmetric expression of the laterality genes became remarkable at Hensen’s node by WISH with NBT/BCIP staining (Fig. 3 and *SI Appendix*, Fig. S3). This classical method has been generally utilized in previous studies to examine the expression of the laterality genes (4, 6, 27, 36–38), however, its sensitivity of RNA detection is relatively lower than the latest techniques such as HCR (28) and Spatial transcriptomics (39). These higher sensitivity/resolution techniques could help to examine the spatiotemporal gene expression pattern in higher resolution and to reveal unknown mechanism(s) of gene regulation in the cellular flows.

Our experimental approaches demonstrated that mitotic arrest maintained the cellular flows despite diminished PS formation and showed initial bilateral movements that later transitioned to R dominance and a single vortex. These results suggest that PS formation is not crucial for initiating the LR laterality in the cellular flows. Our data support the model in which the LR asymmetry in amniote gastrula is already set up, as detected by the cellular flow patterns, prior to when the node-dependent LR patterning kicks in.

If the bilateral cellular flows were symmetric, the three distinct midline axes (AM, BM, and FM, shown in Fig. 4 and *SI Appendix*, Fig. S4) were expected to overlap completely. At the onset of the cellular flow, there was a significant difference in positions for the three midlines. The reason for the high variation is currently unknown but perhaps due to the flow not being fully developed in the beginning (first few hours). The angular separation between the midlines starts reducing as the flow becomes stable as PS continues to extend, but a small difference persists. These temporal variations in the FM could be an important factor that gives rise to the initial LR asymmetry in the bilateral flows. Interestingly, the AM and PS maintain a linear structure during extension despite the LR imbalanced cellular flow (Figs. 1 and 3, *SI Appendix*, Fig. S3 and Movie S1) (22, 40). Our previous study has shown that the authentic PS extends regardless of aberrant flow patterns caused by ectopically induced Vg1 (an axis-inducing morphogen) and secondary PS formation (29). These data support each other and imply that the bilateral rotational cellular flow is not necessarily responsible for PS extension. Rather, the PS is capable of extending by internal mechanism(s), such as convergent extension within the PS (18, 33, 34). Our results imply that the emergent flow mechanics that arise from collective cell movements break LR symmetry and impart LR laterality much earlier than previously expected.

Another unpredicted finding of our study is the R dominance of the flows at later stages during PS extension. The signature of the R dominance was seen readily in the control embryos (Figs. 2 and 4 and *SI Appendix*, Figs. S7–S10). In the mitotic arrest case, the cellular flows are highly asymmetric (nonbilateral). The absence of the PS structure may remove or reduce mechanical stability. Consistent with this idea, in some cases, the flows started to develop bilateral-type movements but merged on one side. In all cases with mitotic arrest, this merging flow was always toward R by forming a single vortex in our data (N = 4). This implies that the R dominance already present in control embryos is being further amplified in the mitotic arrest cases. It remains to be explored how the preferred R dominance is induced and or patterned.

Two models have been proposed for establishing the LR laterality in the chick embryo (8, 41). The classic model is that the LR laterality is set up in the blastoderm by localization of maternal determinant before gastrulation and each LR side develops by following the preset pattern(s) (41). The recent model is that molecular signaling(s) from the midline structures, including the PS, initiates and patterns the LR laterality (8) based on extensive genetic and molecular studies (6, 27, 42). Our present study with quantitative biophysical analyses has revealed that the LR asymmetry in the bilateral cellular flow appears earlier than remarkable asymmetries in the expression of the LR regulatory genes at the Hensen’s node (Fig. 3 and *SI Appendix*, Fig. S3). Further, the R dominance in the flow pattern was preserved even though the PS, at the anterior end of which Hensen's node arises, was diminished (Fig. 5). These data suggest that the LR laterality in the cellular flow is unlikely to depend on the node-driven LR patterning signaling(s). It is currently unknown how the cellular flow is induced and how the LR asymmetry in the bilateral cellular flow is patterned. However, the chirality of movement in mammalian cells has been extensively studied in vitro and appears to be intrinsic and readily scaled up to the level of tissues in bioengineered multicellular systems (43–52). Relevant phenomena with the chirality of cytoskeletons have been identified in invertebrates at early developmental stage prior to LR-organizer formation and play important roles in LR-body patterning (53–56). These previous studies imply that the cellular chirality, exhibited by their movement and cytoskeleton, may be evolutionarily involved in LR-body patterning (57, 58). In the chick embryos at the prestreak stage, several morphogens (e.g. Wnts and Vg1) are already expressed in the embryonic disc prior to and at the onset of cellular flows. Whereas these morphogens are known as axis-inducing factors (59), their roles on the large-scale cellular flow, particularly in amniotes, remain to be determined. We have previously demonstrated the ectopic introduction of Vg1 modulates the cellular flow pattern (29). These morphogens would regulate the cellular chirality, generating the LR asymmetry in the bilateral cellular flow. Further studies examining any LR asymmetries of these morphogens and/or the response in single cells may reveal undefined mechanism(s) of the LR laterality of the cellular flow, such as the early symmetry breaking.

While large-scale cellular flows during gastrulation are evolutionarily conserved (60–62), the LR laterality of the cellular flows remains largely unexplored in most species. Chick embryos begin the cellular flow with the bilateral rotating pattern prior to and during early PS extension and then shift to a lateral movement toward the PS as the ingression takes place at the midline for germ layer formation (31, 32). We have previously reported that the midline structures, including the PS, are required for both LR asymmetry and the ipsilaterality of the lateral cell movements, which subsequently occur after HH3 following the polonaise movements (19). The current study has demonstrated that mitotic arrest diminished PS formation but the LR laterality in the bilateral rotating cellular flow can start (Fig. 5 and *SI Appendix*, Fig. S12). These findings suggest that the role of the midline structures for the laterality/ipsilaterality of the cellular flows would switch in cellular flow patterning the early and later developmental windows of the large-scale cellular flow.

Whether the LR laterality of the cellular flow is related to the later genetically programmed LR patterning remains to be determined. The asymmetric expression of the LR regulatory genes initiates at the Hensen’s node (also termed the “node” in mice, Fig. 3) (25–27). In contrast, the Hensen’s node in chick gastrula has short primary cilia instead of the motile cilia (63, 64). These insights suggest that the model of the cilia-regulating gene expression may not be applicable in all amniotes, and alternative models have been proposed (20, 63, 65). Further studies, for example, disruption of the asymmetric counterrotating cellular flow, will clarify the role of the cellular flows for the genetically programmed LR patterning. Our study would provide an alternative view for symmetry breaking and provides hints for understanding the establishment of LR laterality and bilateral body patterning.

## Materials and Methods

### Embryo Isolation and Culture Condition.

We obtained fertilized eggs (White Leghorn; *Gallus Gallus domesticus*) from Petaluma farms in California. From unincubated eggs, prestreak stage embryos [stage X to XII (24)] were isolated in Tyrode’s solution (final concentration: 137 mM NaCl, 2.7 mM KCL, 1 mM MgCl2, 1.8 mM CaCl2, 0.2 mM Ha2HPO4, and 5.5 mM D-glucose, pH 7.4). The chick embryos were subsequently cultured until HH3 for about 14 h at 37 °C, by using the in vitro culture method termed “New culture” (66) as previously described (29, 40, 66, 67). After electroporation, the embryos with reagents (described as below) were live-imaged on a vitelline membrane stretched around a glass ring according to the New culture method as previously described (29).

### Electroporation.

Isolated embryos from the yolk were transfected with control-oligo DNA (5’-CCTCTTACCTCAGTTACAATTTATA-3) conjugated with Lissamine (GENE TOOLS) using an electroporator (Nepagene) with 3 pulses of 2.4 to 3.8 V, 50 ms duration, 500 ms interval, and platinum electrodes. The DNA solution delivered to the epiblast contained 0.1% fast green (final 0.02%), 80% glucose (final 4%), and 1 mM control-oligo.

### Aphidicolin Treatment.

Aphidicolin (SIGMA, A0781), dissolved in DMSO (SIGMA, B23151), was added to Tyrode’s solution to a final concentration of 100 μM. The isolated embryos were soaked in Tyrode’s solution with either 0.3% DMSO (control), or 100 μM aphidicolin for 15 min at 37 °C. Embryos were then cultured using the New culture method at 37 °C.

### BrdU Assay.

Embryos were soaked in Tyrode’s solution containing BrdU (final concentration; 0.1 mM, Thermo Fisher, B23151) with or without aphidicolin (SIGMA, A0781) at 37 °C for 15 min, and cultured for 16 h at 37 °C (BrdU was incorporated for 16 h). Embryos were then fixed in 4% paraformaldehyde (PFA, Electron Microscopy Sciences)/PBS for 30 min at room temperature (RT). Embryos were then washed with PBS to remove PFA, and unincorporated BrdU, and incubated with 1 M HCl for 1 h at RT to denature DNA. BrdU signal was detected by immunofluorescence staining with anti-BrdU antibody (1: 200, Millipore, MAB3424).

### Whole-Mount In Situ Hybridization (WISH).

The detailed ISH protocol was described in previous studies (19, 29). The cultured embryos were fixed in 4% PFA at 4 °C, and washed in PBST for 5 min in 3 times. After a sequential methanol replacement from 25% to 100% and a subsequent rehydration to PBST, the embryos were treated with proteinase K solution (final concentration: 0.5 mg/mL)/PBST for 1 min at RT. The reaction was neutralized by glycine (2 mg/mL)/PBST and washed in PBST for 5 min in two times. After 2 h of prehybridization in Solution I (final concentration: 1% SDS, 5xSSC pH4.5, 50% formamide) with yeast tRNA (final concentration: 50 μg/mL, MilliporeSigma, MA) and heparin (final concentration: 50 μg/mL, MilliporeSigma, MA), the embryos were hybridized with RNA-probes at 70 °C overnight [probes; *Brachyury, Sox3* (a gift from Dr. Raymond B. Runyan), *Slug, Lefty, Nodal, Shh*]. Next day, the buffer was replaced to new Solution I, and the embryos were washed for 30 min two times. Next, washed in Solution III (final concentration: 50% formamide, 2XSSC pH4.5) for 30 min two times, followed by washing in TBST at RT, 3 times for 5 min each and blocking with 10% sheep serum in TBST at RT. Anti-DIG antibody (final concentration: 1/2,000, Roche, MA) was reacted with the embryos at 4 °C overnight. Then, the following day, embryos were washed in TBST for 1 h, 5 times at RT. After treatment with NTMT (final concentration: 100 mM of NaCl, 100 mM of 50 mM MgCl2, 0.1% Triton) twice for 5 min, color development was performed with NBT/BCIP solution in NTMT (4.5μL of NBT and 3.5μL of BCIP in 1 mL of NTMT). All ISH experiments were parallelly carried out with control embryos to manage the appropriate time of color development.

### Imaging of the Samples.

Live-imaging of the fluorescently labeled chick embryos was performed at 37C° on either microscope, the Widefield Epifluorescent inverted microscope (Nikon Ti inverted fluorescent Microscope with CSU-W1 large field of view, UCSF Nikon imaging center) connected by ANDOR iXon camera or the TIRF/Spinning Disk microscope (Nikon Ti inverted fluorescent Microscope with CSU-22 spinning disc confocal, UCSF Nikon imaging center) connected with Prime 95B Scientific CMOS camera. After live-imaging (29), these samples were subsequently subjected to ISH and imaged by the Leica MZ16F with DFC300 Fx camera and FireCam V.3.4.1 software. BrdU-stained samples were imaged on Ti2 inverted fluorescence microscope connected by the Crest LFOV spinning disk confocal at UCSF Nikon imaging center.

### Image Processing.

Time-lapse imaging datasets from the microscope were opened in the ImageJ (or Fiji) software and split into two channels: brightfield and fluorescence. The brightness and contrast settings of the fluorescence time-lapse images were optimized using suitable thresholds to reduce the noise and enhance the ability to clearly resolve the bright fluorescent markers against a dark background. In the time-lapse sequence, the cellular movements in the embryo only start after a short time delay. For our biophysical motility analysis, we only consider those images after the start of cell movements, this starting point would correspond to the zero time (t), or t = 0 in our analysis. In all the datasets, after determining the starting time, we analyze time-lapse images corresponding to the first 10 h of early development. This helps make standard time-point comparisons between the different experimental datasets presented here.

### PIV.

PIV is an experimental technique to quantify fluid flow-fields. This nonintrusive technique is traditionally based on optical time-lapse imaging of tracer particles that are typically illuminated by a high-intensity light source. Next, the time-lapse image sequence is analyzed further and processed using computational algorithms to obtain a spatiotemporal map of flow velocity vectors. Here, we have utilized PIV analysis techniques on the time-lapse images of fluorescently tagged cells in the embryo to quantify their movements and speeds over time. We employed the MATLAB-based PIVlab package for our data analysis (21) (*SI Appendix*).

### Time Averaging of PIV Datasets.

The PIV analysis by default provides instantaneous velocity vectors that are calculated between two time-points (here this timescale is 3 min). In our datasets, the cell movements over short timescales of 3 min are very small and the time-resolved transitions in the datasets are inherently noisy due to spatial and temporal fluctuations. Hence, we calculated the time-averaged measurements of the key cell movement parameters (speeds, vorticity) over longer timescales. We found that these time-averaged measurements were significantly better for both detailed quantification and interpretation of asymmetry in the bilateral cellular flows. We carried out three different durations of time averaging – i) 10 h, ii) 5 h, and iii) 1 h. This enables interpretation and comparison of bilateral cellular flows at different timescales in the datasets. i) Time averaging over 10 h gives an overall picture of the cellular flows during early development. ii) Time averaged quantification over 5 h enables us to distinguish between the first half and second half of the process. iii) Time averaged quantification over 1 h enables us to carefully study questions about cellular flows such as spatial and temporal origins, changes and transitions, and dominance of L-R asymmetry spanning 10 time-points distributed over every 1 h. The two key parameters that we use to quantify and characterize cellular flows are speed and vorticity, and we have studied their variation over time during the process of development.

### Speed Plots.

The speed at every spatial point at a given time is calculated as the vector magnitude of the x and y components of the velocities in PIVlab. The cellular flow speeds are plotted based on the thresholds (described below) at each time point for all the datasets using a heatmap color scheme continuously varying from low speeds (blue) to higher speeds (red).

### Streamline Plots.

Streamlines are the instantaneous and simplified representations of fluid flow directions or paths. Each streamline is tangent to the velocity vector of the fluid flow at every point along its path. They are very helpful in visualizing and analyzing fluid flow. The flow parameters such as vorticity can be better interpreted by comparison with the streamlines. Here, the streamlines were plotted manually with appropriate line width and color. Selecting the “draw streamlines” option in PIVlab and clicking on the vectors automatically generates the most useful streamlines.

### Vorticity Plots.

Vorticity is a vector quantity commonly used in fluid dynamics to quantify the local rotation of a fluid element. Mathematically, the vorticity is calculated as the curl of the fluid velocity. It provides important insights into the rotational regions in the cellular flows, and this rotation can either be clockwise or counterclockwise. High vorticity regions indicate strong local rotation, whereas low vorticity points toward weak rotation. Vorticity plots were generated for all datasets in PIVlab using a heatmap color scheme continuously varying from high clockwise vorticity (red) to 0 (white) to high counterclockwise vorticity (blue).

### Biophysical Quantification of PIV Datasets.

In addition to the default plotting of PIV velocity flow fields and their derived quantities such as speed and vorticity, we further extracted quantitative information to characterize the cellular flows in different datasets as described below (*SI Appendix*).

### Midlines.

We defined three midlines in the control datasets: AM (defined by the biological structure), BM, and FM (defined by the cellular flows). The AM is the line passing through the center of PS (Fig. 1*A*). The bilateral flow establishes two counterrotating vortices in L and R regions. The line drawn perpendicular to the midpoint of the line joining the vortex centers is defined as BM (*SI Appendix*, Fig. S4*A*). A line drawn parallel to the flow streamlines passing through the midpoint of the vortex centers is defined as the FM (*SI Appendix*, Fig. S4*A*).

### Vorticity and Speed Thresholds for Quantification.

In each control sample dataset, for every hourly averaged speed plot, the maximum speed (V_max_) value was identified. Based on this V_max_ value, three thresholds of 30%, 50%, and 70 % were calculated (*SI Appendix*, Table S3). Here, 30% threshold means velocity vectors with the magnitude of 30% of V_max_ (i.e. V_max_*0.3) were retained, and similarly for 50% and 70%. Similarly for vorticity, the maximum vorticity (magnitude) value was obtained over 1 h. Again, based on this maximum vorticity magnitude, the three thresholded values, 30%, 50%, and 70%, were obtained (*SI Appendix*, Table S4). Speed and vorticity values that are higher than these thresholds were considered for quantifying the areas on the left (A_L_) and right (A_R_) regions. This approach enabled a rigorous quantification of cellular flows at different magnitudes (with varying thresholds in speed and vorticity) and the comparison between L and R regions over time (*SI Appendix*, Figs. S7–S10).

### Statistical Test.

Statistical analysis was done using the nonparametric Wilcoxon test in JMP Pro 17.0.0. The obtained *P* values are mentioned in the relevant Fig. legends and in supplementary tables (*SI Appendix*, Tables S5 and S6).

## Supplementary Material

Appendix 01 (PDF)

Movie S1. **Cellular flows reveal L-R asymmetry during early chick embryo development**. The top left panel is a brightfield imaging time-lapse video of early chick embryo development, revealing the development of the Primitive Streak (PS). The top right is a Flowtrace visualization video showing large-scale bilateral cellular flows or ‘polonaise movements’, the cells are tagged with fluorescent labels. The bottom left is a heatmap video of cellular speeds, calculated using the Particle Image Velocimetry (PIV) technique. The bottom right is a heatmap video of vorticity (quantification of rotation), calculated using the PIV technique. The white and black arrows in the bottom panels (left and right respectively) indicate the direction and magnitude of the local velocity field of cellular motion. In the bottom panels, towards the end of the video, we see a Right-side dominance of the cellular flows, indicating a deviation from Left-Right (L-R) symmetry. All videos are synchronized and correspond to the same dataset with a total duration of 10 hours, and the timestamp represents hours and minutes.

Movie S2. **Cellular flows in cell-division inhibited chick embryos**. The top left panel is a brightfield imaging time-lapse video of a cell-division inhibited chick embryo, where the Primitive Streak (PS) does not form. The top right is a Flowtrace visualization video showing a Right-vortex dominated cellular flow, the cells are tagged with fluorescent labels. The bottom left is a heatmap video of cellular speeds, calculated using the Particle Image Velocimetry (PIV) technique. The bottom right is a heatmap video of vorticity (quantification of rotation), calculated using the PIV technique. The white and black arrows in the bottom panels (left and right respectively) indicate the direction and magnitude of the local velocity field of cellular motion. In the top right panel, and bottom panels, from the middle of the video until the end, we see only one Right-vortex dominated cellular flow. All videos are synchronized and correspond to the same dataset with a total duration of 10 hours, and the timestamp represents hours and minutes.

## Data Availability

All experimental datasets, plot data, and codes generated in this study have been deposited in a Zenodo repository (68). The data was analyzed using the open-source PIVlab package in MATLAB: https://www.mathworks.com/matlabcentral/fileexchange/27659-pivlab-particle-image-velocimetry-piv-tool-with-gui (69); https://pivlab.blogspot.com/ (70).
